# Understanding care-seeking of pregnant women from underserved groups: a systematic review and meta-ethnography

**DOI:** 10.3389/fpubh.2025.1683740

**Published:** 2025-11-20

**Authors:** Tisha Dasgupta, Hannah Rayment-Jones, Gillian Horgan, Yesmin Begum, Michelle Peter, Sergio A. Silverio, Laura A. Magee

**Affiliations:** 1 Department of Women and Children’s Health, School of Life Course and Population Sciences, King’s College London, London, United Kingdom; 2 The ENGAGE Study Patient and Public Involvement and Engagement Advisory Group, King’s College London, London, United Kingdom; 3 Department of Psychology, Institute of Population Health, University of Liverpool, Liverpool, United Kingdom

**Keywords:** health care-seeking, maternity care, high-income countries, women, health inequality, marginalized groups

## Abstract

**Background:**

Delayed or reduced antenatal care use by pregnant women may result in poorer outcomes. ‘Candidacy’ is a synthetic framework which outlines how people’s eligibility for healthcare is jointly negotiated. This meta-ethnography aimed to identify – through the lens of candidacy – factors affecting experiences of care-seeking during pregnancy by women from underserved communities in high-income countries (HICs).

**Methods:**

Six electronic databases were systematically searched, extracting papers published from January 2018 to January 2023, updated to May 2025, and having relevant qualitative data from marginalized and underserved groups in HICs. Methodological quality of included papers was assessed using the Critical Appraisal Skills Program. Meta-ethnography was used for analytic synthesis and findings were mapped to the Candidacy Framework.

**Results:**

Studies (*N* = 51), with data from 1,347 women across 14 HICs were included. A total of 12 sub-themes across five themes were identified: (1) Autonomy, dignity, and personhood; (2) Informed choice and decision-making; (3) Trust *in* and relationship *with* healthcare professionals; (4) Differences in healthcare systems and cultures; and (5) Systemic barriers. Candidacy constructs to which themes were mapped were predominantly joint- (navigation of health system), health system- (permeability of services), and individual-level (appearances at health services). Mapping to Candidacy Framework was partial for seven sub-themes, particularly for individuals with a personal or family history of migration. The meta-ethnography allowed for the theory: ‘Respect, informed choice, and trust enhances candidacy while differences in healthcare systems, culture, and systemic barriers have the propensity to diminish it’.

**Conclusion:**

Improvements in antenatal care utilization must focus on the joint (service-user and -provider) nature of responsibility for care-seeking, through co-production. We suggest two additional Candidacy Framework constructs: ‘intercultural dissonance’ and ‘hostile bureaucracy’, which reflect the multi-generational impact of migration on healthcare utilization and the intersection of healthcare utilization with a hostile and bureaucratic environment.

**Systematic review registration:**

https://www.crd.york.ac.uk/PROSPERO/view/CRD42023389306, CRD42023389306.

## 1 Introduction

Routine antenatal care is a globally recommended public health service enabling healthcare professionals (HCPs) to provide essential information, counseling, maternal and fetal assessments, and encourage use of maternity services (1, 2). Delayed or reduced antenatal care use, in both high- (HICs) and low−/middle-income countries (LMICs), is linked to adverse pregnancy outcomes, including stillbirth (3), and neonatal morbidity (4).

Research on maternity care-seeking has largely focused on LMICs, where barriers are often financial, geographic, or linked to knowledge gaps beliefs about the importance of maternity care (5). In contrast, many HICs offer free healthcare at point of access, yet barriers remain. Even with structurally accessible services, uptake remains low in certain communities, including those of lower socio-economic status (SES), minority ethnic groups, sexual minorities, and people living with disabilities (6–8).

A recent meta-synthesis of qualitative studies in HICs highlighted multiple barriers (e.g., socio-demographic disadvantage, system navigation, lack of tailored care, frequent carer changes) and facilitators (e.g., positive pregnancy attitudes, good HCP interactions, social support) (9). Furthermore, the pandemic introduced additional barriers to care-seeking (i.e., social isolation, personal infection risk (10), poorer mental well-being (11), continuing restrictions for perinatal populations after lockdowns (12), and navigating healthcare service reconfigurations (13, 14)), with experiences of care being reported more negatively with poorer mental health outcomes (11–18).

‘Candidacy’ refers to people’s eligibility for accessing healthcare. It was developed to explain unequal access to healthcare, despite universal health coverage, and to go beyond simple measurement of health utilization, particularly by marginalized groups (19). The theoretical framework of ‘candidacy’ refers to healthcare access as negotiated jointly between service-user and healthcare system. It describes a dynamic process, subject to external influences, from people and their social context, as well as available resources and service structure (19). There are seven constructs of: identification, navigation, permeability of services, appearances at health services, adjudication, offers and resistance, and local production of candidacy (19). This framework was chosen to guide this systematic review in order to establish a theory driven structure, moving beyond simply identifying barriers to rather describe the process by which access is negotiated. This is crucial in perinatal contexts where marginalised women face layered challenges related to stigma, institutional bias, and bureaucratic hurdles in the process of accessing and engaging with care (20). Mapping systematic review results to the constructs of the Candidacy framework allowed comparison of evidence across diverse populations in a coherent way. As used previously in healthcare research, this framework lends itself well to understanding the latent factors influencing care-seeking among marginalised groups, for which it was first developed (21–26). The framework was employed within the systematic review in order to strengthen analytical rigor and improve the potential to generate actionable insights.

The aim of this systematic review and meta-ethnography was to synthesize qualitative evidence from HICs, to identify – through the lens of candidacy – factors affecting experiences of care-seeking during pregnancy, by women and birthing people from underserved communities. We expand on previous work by focusing solely on underserved groups known to face additional barriers to care access, utilization, and engagement.

## 2 Methods

This review was registered with PROSPERO [CRD42023389306] and adheres to the PRISMA 2020 statement (Supplementary Table S1) (27).

### 2.1 Inclusion and exclusion criteria

The PEO (Population, Exposure, and Outcome) framework was used to formulate the search strategy as per the research aim (Supplementary Table S2).

- Population: Women and/or birthing people planning pregnancy, pregnant, or postpartum, in an HIC setting (as classified by the World Bank, 2024), and from an underserved community, defined as individuals or groups with one or more social risk factors, which may have resulted in them being systematically excluded or denied full opportunity to participate in economic, social, or civic life. Social risk factors were as expansive as possible, including: young or advanced maternal age; single mothers; low SES; any group identified as a minority within the study setting (e.g., ethnicity or sexual orientation); refugee, or asylum-seeker; facing homelessness, victim/survivor of domestic abuse; living in a deprived area; having a diagnosed mental health condition or learning disability; physical disability or chronic illness; substance abuse; and not speaking the language local to the country in which the healthcare was provided.
- Exposure: All routine antenatal and intrapartum care, comprising planned care before and during labor and birth, to optimize outcomes for mothers and babies, as defined by WHO guidelines (WHO, 2016). This care includes the minimal number of planned antenatal care appointments, health promotion activities (such as advice on healthy diet and exercise), urine and blood tests (such as to screen for anemia), vaccination and supplementation (such as with iron), monitoring of fetal wellbeing, additional care for women and/or birthing people at higher risk, and care provided for labor and birth (such as for progress in labor and skilled birth attendance). The setting could be within hospitals, the community, or at home.
- Outcome: Care-seeking experiences, including health knowledge, behaviors, perceptions, and healthcare utilizations.

Study designs included: descriptive, exploratory, and interpretive qualitative studies; ethnographies; and observational or mixed-methods studies (including surveys with open-ended questions) where qualitative data had been formally analyzed and presented (24). Studies were published between Jan 2018–23, updated to May 2025, and only considered if published in English-language. Studies of postnatal care were excluded due to its variation between countries, and its fragmented nature, often spanning services in primary through to quaternary care settings.

### 2.2 Search strategy and selection

Electronic databases of SCOPUS, MEDLINE, EMBASE, CINAHL, Global Health, PsychINFO, and MIDRIS were systematically searched for articles published between 1 January 2018 and 1 January 2023, updated to May 2025 (28). For details of the search terms and keywords used, see Supplementary Table S2.

Duplicate references were removed using Mendeley reference manager software, and citations were uploaded to Rayyan (29), a web-based tool for conducting systematic reviews. At least two members of the study team (TD, HRJ, GH, SAS, LAM) independently screened each record, by title and abstract, followed by full-text review. Regular discussions were held to resolve by consensus any disagreements in screening decisions.

### 2.3 Data extraction

Data extraction was randomly allocated to one of two reviewers (TD, GH), with 20% of included studies extracted independently by both reviewers to check between-reviewer reliability. A bespoke Microsoft Excel spreadsheet was used to abstract study characteristics (i.e., title, reference, publication year, study setting, aim, participant inclusion criteria, intersectional approach, data collection and analytic methodologies), and any impact of the pandemic on care-seeking. Regular discussions were organized to discuss any disagreements, and to collaborate on the creation of a consolidated set of themes with consistent labels.

### 2.4 Quality assessment

The Critical Appraisal Skills Program (CASP) was used to assess the quality of included studies (30) across 10 items: clearly-stated objective, appropriateness of using qualitative study design, justification of research design, recruitment strategy, data collection method, author reflexivity, ethical considerations, data analysis method, clear findings, and value of the findings. CASP does not assign a score, but for ease of interpretation, we assigned points to answers for each checklist item: 0 points for ‘No’, 1 for ‘Cannot tell’, and 2 for ‘Yes’.

### 2.5 Data synthesis

**Figure 1:**
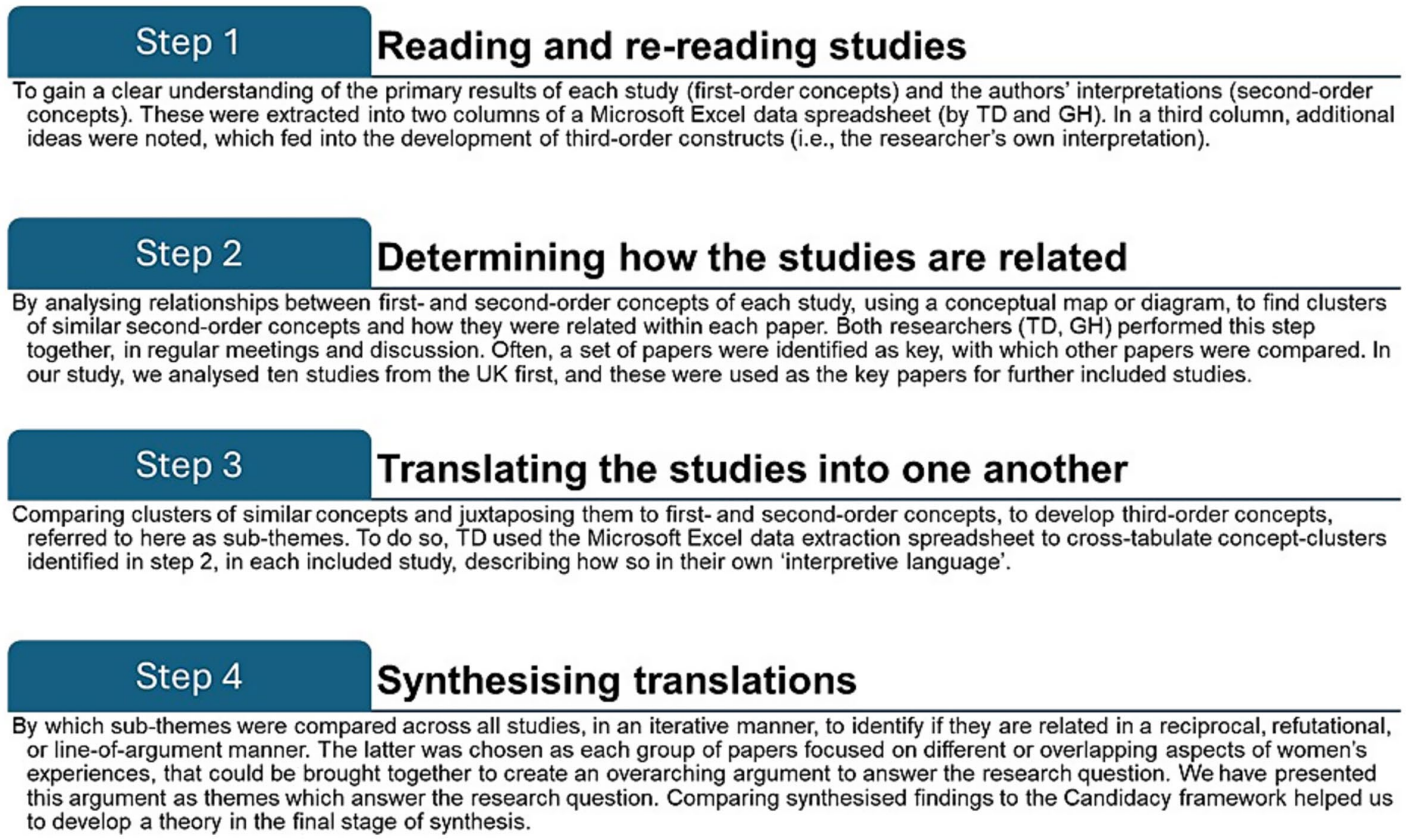
Steps of meta-ethnography.

Meta-ethnography (31) was employed for analytic data synthesis, which is a particularly useful approach when addressing complex questions, as it enables comparison between and across published studies, and creates higher-order themes which can be newly-interpreted, based on the wealth of integrated data (31, 32). Syntheses can be reciprocal (studies are similar to each other and shared themes across the studies are summarized), refutational (studies refute each other and themes are juxtaposed against each other), or ‘line of argument’ (studies interpret the same phenomenon but from different aspects, the synthesis creating a whole greater than the sum of its individual parts) (32). Typically, there are four main steps, as employed by other researchers (33, 34), outlined in Figure 1, (32–34).

Intersectional approaches in included studies were considered to compare participant groups. Synthesized themes and sub-themes were mapped to one or more of the seven components of the Candidacy Framework (19), as in Table 1, to determine how the identified factors influence eligibility of candidacy; with the weight of each theme contributing to candidacy was calculated.

**Table 1:** Candidacy framework constructs.

Candidacy framework factor	Definition	Individual-level	Health system-level
Existing factors			
Identification	Self-acknowledgement of necessity of medical attention for symptoms	●	-
Navigation	Learning about and negotiating services	●	●
Permeability	Ease with which people can use services	-	●
Appearance at health services	Individuals’ ability to articulate their need for care and assert their candidacy	●	-
Adjudication	Health care-providers’ judgments dictating progression of individuals’ candidacy	-	●
Offers & resistance	Declining to accept care offers, medications or referrals	●	●
Local production of candidacy	Local factors influencing candidacy, including availability of resources and long-term patient-provider relationships	●	●

## 3 Results

### 3.1 Search and selection

Of 3,098 records identified, 2,493 underwent title and abstract screening, 68 underwent full-text review, and 45 were included (35–79) (see Supplementary Figure S1). An updated search to May 2025, identified six additional records for analysis (80–85).

### 3.2 Description of included studies

The 51 included studies provided data from 1,347 service-users. Studies were published between 2018 and 2025, from 14 countries, most commonly the USA (*n* = 13 studies) (36–38, 46, 52, 53, 57, 61, 64, 67, 68, 80) and the UK (*n* = 13) (58, 70–79, 84, 85), followed in frequency by Australia (*n* = 5) (43, 49, 56, 66, 82), Norway (47, 50, 69), Denmark (45, 62, 63), Sweden (35, 48, 51), Switzerland (41, 42), Netherlands (40, 65), New Zealand (44, 81), Canada (39), Germany (60), Israel (55), Russia (83), and Saudi Arabia (59). The most common data collection method was in-depth interviews (*n* = 34) (35, 39–41, 43, 45, 48–51, 53–60, 62–64, 67–69, 71, 75, 76, 78–83, 85), followed by focus groups (*n* = 13) (36–38, 42, 44, 46, 47, 52, 61, 65, 70, 72, 74), surveys with open-ended questions (66, 73, 77, 84), and ethnographic observations (70). Some studies used multiple methods (*n* = 6) (36, 44, 46, 47, 70, 74). Most studies utilized thematic analyses (*n* = 31) (37, 38, 41, 42, 44, 47, 52, 54–59, 61, 63, 65, 66, 68–71, 73, 74, 77–82, 84, 85); others used framework analyses (*n* = 5) (40, 64, 72, 75, 76), content analyses (*n* = 5) (35, 48, 51, 60, 67), grounded theory analysis (*n* = 4) (36, 39, 46, 53), interpretative phenomenological analysis (49, 83), systematic text condensation (45, 50), qualitative comparative analysis (43, 57), or interpretive description analysis (68). Two studies (50, 66) evaluated the impact of the pandemic on care-seeking experiences.

Social risk factors included: being migrants, refugees, or asylum-seekers (*n* = 18) (35, 36, 42, 45–48, 50, 51, 56, 60, 65, 67–70, 74, 75, 79); being racial, ethnic or religious minorities (*n* = 9) (37–39, 44, 52, 54, 55, 72, 81); having low SES (*n* = 8) (71–73, 76, 79–82); not being able to speak the local language (*n* = 7) (41, 51, 54, 57, 58, 60, 66, 85); having previous interaction with social services or child protection services (*n* = 4) (62, 63, 76, 84); having substance abuse issues (*n* = 4) (53, 55, 62–65, 84, 86) having learning, intellectual, or physical disability or impairment (*n* = 3) (77, 81, 84); being a victim of domestic abuse or intimate partner violence (*n* = 3) (43, 79, 84); being a young mother (*n* = 3) (79, 81, 84); living in a rural setting (*n* = 3) (52, 82, 83); having missed or delayed antenatal care (*n* = 2) (59, 64); experiencing homelessness (*n* = 2) (78, 84); or having transgender pregnancy (*n* = 1) (81). Nine studies described participants with medical complexity such as preterm birth and gestational diabetes (36, 38, 40, 48, 52, 56, 61–63). For further details, see Supplementary Table S3.

### 3.3 Quality assessment

Study quality was moderate-to-high (Supplementary Table S4). Of a possible score of 20, all studies scored ≥14, as follows (60):14/20 (*n* = 2) (39, 66), 15/20 (*n* = 4) (35, 54, 75, 84), 16/20 (n = 4) (53, 61, 69, 73), 17/20 (*n* = 15) (36, 44, 47–49, 57, 59, 60, 63, 65, 67, 74, 76, 78, 79), 18/20 (*n* = 13) (41, 42, 45, 50, 52, 55, 56, 64, 71, 72, 77, 83, 85), 19/20 (*n* = 5) (37, 43, 46, 58, 80, 81), and 20/20 (*n* = 7) (38, 40, 51, 62, 68, 70, 82). Those highest-scoring studies which did not reach 20/20 often fell short by missing consideration of the relationship between researchers and participants and associated ethical issues.

### 3.4 Analytic synthesis and findings

**Figure 2:**
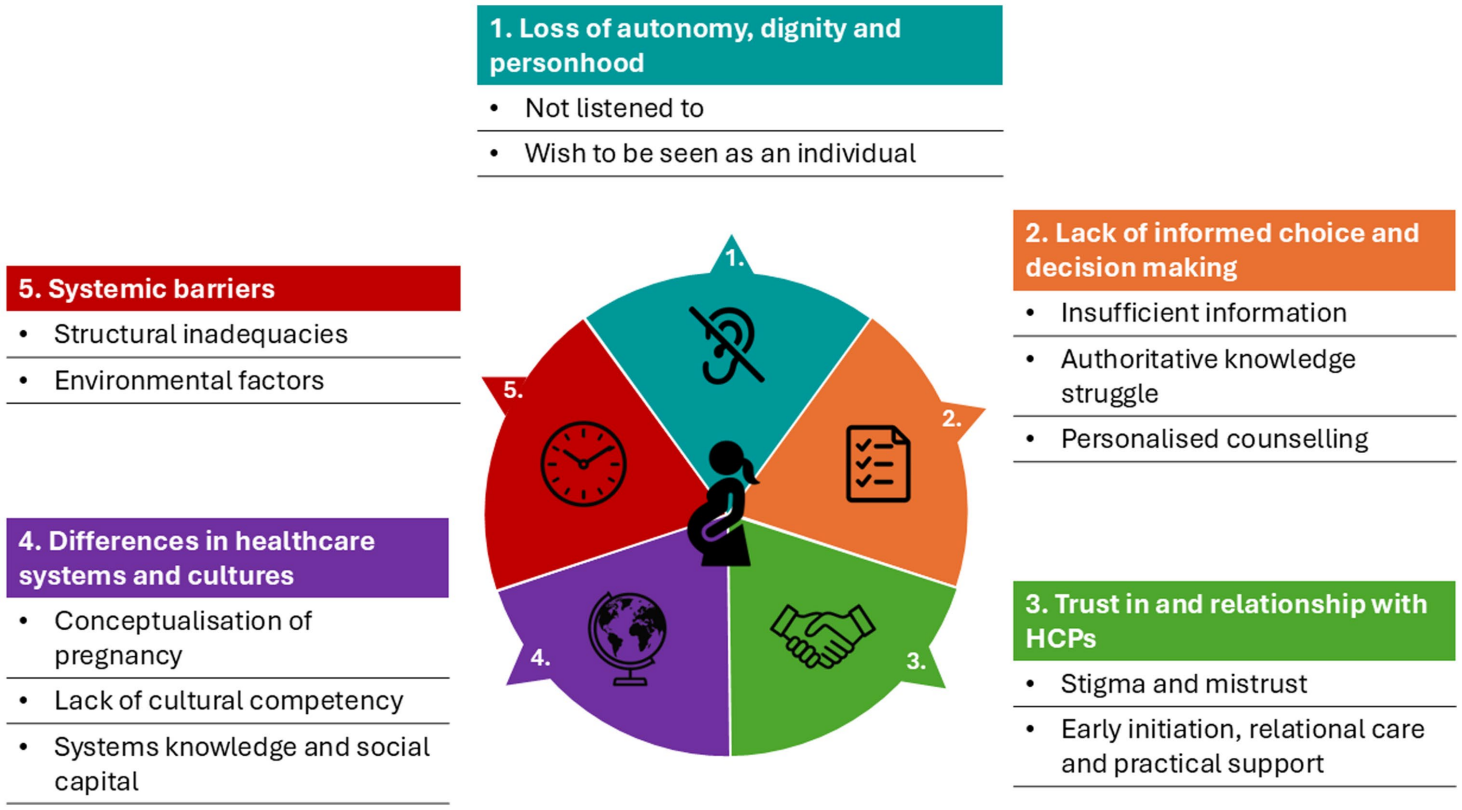
Findings of factors affecting women’s experience of care-seeking. Illustrations created using Chat GPT.

Figure 2 depicts the theory derived from the data: *‘Respect, informed choice, and trust enhances candidacy whilst differences in healthcare systems, culture, and systemic barriers have the propensity to diminish it’.* The 12 sub-themes were grouped into five main themes: (1) Autonomy, dignity, and personhood; (2) Informed choice and decision-making; (3) Trust *in* and relationship *with* HCP; (4) Differences in healthcare systems and cultures; and (5) Systemic factors. Excerpts of text from individual studies are presented in Table 2 to support the synthesised findings (direct participant quotations are in italics), selected to be the most representative of the analytical theme and idea being described.

**Table 2:** Key quotations to support thematic findings.

Theme	Sub-theme	Key excerpts
1. Loss of autonomy, dignity, and personhood	1.1 Not listened to	“*I also think like if you are on Medi-Cal or you are a certain race versus private insurance it makes a difference because I’ve watched the same doctor. He was nice to this little white couple, but a single black woman coming in, even though the father came with me, it was like I did feel like the treatment was different. And it’s like I do feel like if we do not have private insurance they do treat us differently. They cut costs, cut edges, or do not tell us everything at some of these hospitals*” (38) “*… In week 24 I call the ward and say… ‘the baby is not very active’. They tell me ‘you know this is normal at this time of the pregnancy’. I ask them ‘could you just examine me for a few minutes? I can come in’…They tell me ‘no, if you come in, we are only able to listen to the heartbeat, and if it’s fine, that’s it’. I tell them ‘okay then I will not bother. I have a device’. I am able (to listen to the heartbeat)*”
	1.2 Wish to be seen as an individual	“*[The specialist midwife] actually thought about me as a person, rather than just being a pregnant mum*” (79) Worried that their midwife would judge them on their decision to continue an unplanned pregnancy, a young couple had been positively surprised by her non-judgmental attitude (63).
2. Lack of informed choice and decision-making	2.1 Insufficient information	“*I had asked them what’s the infection that [my baby] has; I wanted them to explain it better to me …. They made me feel really frustrated that they were not really explaining it to me. I tried asking the nurse that was in the NICU and they did not know how to explain it to me … I do remember asking them and asking them*” (46) “*We should be able to just trust that our GP will give us…these are all your options, this is everything you can do. But if you do not have the capacity or maybe the healthy mistrust to check that for yourself, then that’s the first barrier, I guess*” (82)
	2.2 Authoritative knowledge struggle	Advice from friends was generally perceived to be trustworthy. Thus, advice from friends would sometimes precede and annul the necessity of seeking medical advice. This also seemed to be the case when this advice conflicted with advice from maternity care providers (45). “*I guess knowing what I should and should not be doing has been challenging because you read a lot of things and sometimes the information is conflicting and then because I have access to the research, I look up the research, but even the research is inconclusive on most things*” (49)
	2.3 Personalized counseling	“*…the midwives maybe should advise more the clients, the patients, because at least in my culture, in my country, when a doctor says something or when a nurse says something, when a midwife, it’s more trustful. And the patient take it more seriously than they will take in information on internet or in a leaflet*…” (74) In juxtaposition to the majority of patients who expressed a desire to know their personalized preterm birth risk, prenatal care providers reported differing disclosure practices and often cited concerns about patients’ reactions as part of the calculus that went into their decision-making (36).
3. Trust *in* and relationship *with* HCPs	3.1 Stigma and mistrust	“*We got a midwife that we experienced as racist so it did not feel right and so, we requested to get a new one… it was like she did not care much about us… she wanted us to go back to our home country and she barely looked at us… We got worried that she did not want to help us… got afraid that she would hurt the child. We arrived a little late, she got extremely angry at us and told us off. She said ‘why did you come, we have other things to do*’” (35) “*I was a bit scared, also because I never hide the fact that I had the abuse I had. I told our midwife. So there was this fear, [midwife saying], okay, she [partner] has this illness, he used to be an addict, right?(…) That they would think, okay, she will become psychotic and become ill, he will have a relapse [into abuse], when they have the baby. I think we both carried that fear*”… Fear of being judged was often shaped by previous negative experiences where parents had felt misunderstood, misjudged, or stigmatized (62).
	3.2 Early initiation, relational care, and practical support	“*I only met the midwife once so far and she already mentioned that I would probably meet another midwife next time. I think women would be more honest if they could build a trusting relationship with their midwife and see the same midwife throughout their pregnancy*” (66) “*And Birth Companions were really lovely actually because they asked me if there’s anything I needed. And I sort of mentioned, well I’m going to need to get a cot. And they, one of them organized, they came round, one of them brought the cot round*” (76)
4. Differences in healthcare systems and cultures	4.1 Conceptualisation of pregnancy	She was comparing two very different systems [of home vs. host country], reflective of differing policy frameworks and representing different user characteristics in willingness and ability to pay for private services. Nevertheless, the difference made her nervous. “*You get curious. ‘Is the baby okay? Is it no okay?’, you know, just waiting for that first ultrasound [in Norway] seems like a very, very long time. You really want to know [how the pregnancy is going]*” (69) Not being offered more/4-D scans or given detailed explanation of scan results led to their unsatisfaction and anxieties. This led some of them to purchase private scans and amniocentesis (75).
	4.2 Lack of cultural competency	Perceived cultural differences between Indigenous and non-Indigenous peoples and subsequent stereotyping affected the ways in which Indigenous patients and health care providers interacted with one another. Indigenous patients’ nonverbal communication style emerged as an important perceived cultural difference that frustrated both doctors and nurses alike (39) “*They [the nurses] kept bringing me ice water, and we do not do that in Mongolia. We drink hot tea, or at least warm water, and more warm foods. I told them about it, but they just always brought it with ice, so I did not feel they were that supportive*” (67)
	4.3 Systems knowledge and social capital	Women suggested that reasons for non- or late attendance at antenatal appointments included misunderstanding and poor knowledge of health-care system norms. “*[In Pakistan] they just go there, straight away. Take a number and sit. And when they call them – they go and tell the doctor what’s going on. And that’s why people do not know about appointments [here], you know, to make them*” (70) One of them reported that her biggest fear was not to be able to communicate with the midwife or the doctor during childbirth. To avoid this kind of situation, she took her 15-year-old daughter as language mediator with her. The birth proved to be very difficult and although the woman tried several times to send her daughter away, the girl refused to leave her mother. Although her presence was perceived as helpful, the mother later worried about the emotional well-being of her daughter [after a traumatic birth] (60).
5. Systemic barriers	5.1 Structural inadequacies	“*I waited two to three hours! And when I saw the doctor and told her about my concerns, she did not care at all. In Arabic, she ignored what I said. She said,” normal, everything is normal,” and she did not give me time to ask questions. She wanted to finish and take in the next patient*” (59). “*None [no reasonable adjustments were provided]. I had to remain in bed because my wheelchair could not fit in the room. Totally removed my independence*” (58, 77)
	5.2 Environmental factors	In addition to the challenges of accessing health insurance, numerous participants discussed delaying prenatal care until the time of birth due to the lack of insurance or the means to pay. As one participant explained: “*There are some people [within the Marshallese community] that say, ‘wait until your stomach hurts and it’s time for you to give birth and you can go because they’ll have to see you either way,’ because that’s the problem, it’s the money*” (37) “*… the nurse assumed I was going to be pregnant a whole lot of times and she suggested right away I should get rid of my child without asking me if I wanted to or not. So, is it based on my race? Is it based because I’m already high-risk of dropping out of high school because all that was told to me? So, they were already determining what I was going to do with and setting limits on me just because of my race. How much of that is influenced on my race? I think all of it*” (38)

N/B, Direct participant quotations are presented in italics.

#### 3.4.1 Theme 1: Autonomy, dignity and personhood

This theme was identified in 16 studies (35, 38, 42, 45, 46, 55, 59, 63, 67, 71, 76–79), and had two sub-themes.

##### 3.4.1.1 Not listened to

Participants expressed they were not listened to, their concerns were dismissed, or they were made to feel unintelligent and judged for asking questions (38). Some attributed this treatment to personal characteristics, such as ethnicity. This led women to hesitate to ask further questions, attending appointments unless absolutely necessary, or engaging with maternity care overall (78). Some were unaware of their rights and the level of care to expect and request. This made women accept poor quality-of-care and discriminatory practices as part of standard maternity care (55).

##### 3.4.1.2 Wish to be seen as an individual

Women wished to be respected and treated as individuals. They valued when effort was made to understand their background and life beyond pregnancy (79); this often had a protective effect on care-seeking and engagement and built capacity for positive parenting and health (76).

#### 3.4.2 Theme 2: Informed choice and decision-making

This theme was identified in 25 studies (36, 37, 40, 43, 44, 46, 48–51, 56–59, 61, 69, 70, 72–74, 76, 79), and had three sub-themes.

##### 3.4.2.1 Insufficient information

Women felt information was inadequate, and lacked justification for recommendations, which left them wanting more control over their care (56). Some studies reported women feeling HCPs’ own bias and perceptions of patients influenced the information they provided, so they offered only the information they deemed would be relevant for the patient (60). Women felt it fell to them to seek-out information (via friends and family, or online sources, often unofficial (75)), and make decisions about which recommendations to follow, although they felt those decisions were seldom fully-informed (40).

##### 3.4.2.2 Authoritative knowledge struggle

Women often faced balancing information from various sources (70). This included differences in care between their home countries and their current healthcare system, between friends/family and HCPs, between care-providers, or between protocols in different hospitals (58, 70).

##### 3.4.2.3 Personalised counseling

Women emphasized the value of personalized counseling by their HCP, peer support from their communities, and HCPs having the right tools to support women and families, such as knowledge of cultural practices (43, 74).

#### 3.4.3 Theme 3: Trust *in* and relationship *with* HCPs

This theme was identified in 25 studies (35, 38–41, 48, 50–53, 58, 62, 63, 66, 68, 71, 76–79), and had two sub-themes.

##### 3.4.3.1 Stigma and mistrust

The underserved populations studied were often already anxious about being pregnant, so trust played a particularly important role in determining if they attended appointments, disclosed their circumstances, or participated in maternity care (58). Many had established mistrust in HCPs and institutions in general, due to prior negative interactions with social care, immigration, or law enforcement (63, 78). Some feared being reported and their child being removed to services, and so they did not engage honestly with maternity HCPs (78). Women reported feeling unfit as mothers, and stigmatized when honest about social risk factors (e.g., prior drug use or homelessness) (53, 68).

##### 3.4.3.2 Early initiation, relational care, and practical support

When maternity care was initiated early and there was relational care, this built trusting relationships with HCPs and facilitated open discussions (66, 78). Women with mental health issues felt more likely to fully disclose during psychosocial assessments, and those with disabilities did not have to reiterate their accessibility requirements at every appointment (66, 78).

Practical support (e.g., with baby food, blankets, or pushchairs), or emotional support when attending social care appointments helped women embrace new motherhood (48, 68). When they were supported in such ways, it enabled women to make long-lasting changes and prevent relapse to pre-pregnancy habits such as substance abuse.

#### 3.4.4 Theme 4: Differences in healthcare systems and cultures

This theme was identified in 21 studies (37–39, 42, 45, 47, 48, 52, 56–58, 60, 61, 69–73, 75), and had three sub-themes.

##### 3.4.4.1 Conceptualisation of pregnancy

Studies emphasized how pregnancy is conceptualized differently by setting. In some countries, antenatal care was described as highly-medicalised, with multiple appointments and ultrasound scans. In other settings, there may be only two or three contacts throughout pregnancy, even though official guidelines and recommendations may suggest more (42, 60). Such differences often concerned mothers who had migrated from one country to another and altered their health literacy and ability to risk-assess their pregnancies. Some women did take on board new opportunities; when given the choice and relevant information, women from minority ethnic communities in the UK expressed a desire to have more home births (72).

##### 3.4.4.2 Lack of cultural competency

Differences in social norms around pregnancy, information shared, standard practice, role of the birth partner or other family members, and religious beliefs, greatly-influenced women’s views of the acceptability of care offered, or even the decision to attend appointments (39, 47, 56). Women felt that HCPs lacked cultural understanding and did not treat them with respect, which led to negative interactions.

##### 3.4.4.3 Systems knowledge and social capital

Migrant women had trouble understanding how to access or use maternity care services in their host country, including when and how to make appointments (37, 47). Many such women lacked social capital, described as playing a protective role, particularly postnatally. Often, they lacked support from wider familial networks during maternity care, and in life generally, to interpret for them if they did not speak the local language (69).

#### 3.4.5 Theme 5: Systemic barriers

This theme was identified in 24 studies (37–39, 42, 44, 47, 48, 51, 52, 55, 58–61, 64, 66, 71, 75–77), and had two sub-themes.

##### 3.4.5.1 Structural inadequacies

Lack of flexibility in scheduling appointments, long wait-times in hospital, and rushed appointments with HCPs, posed barriers to engagement with maternity care (44, 61, 71). Studies reported poor communication between women and HCPs, due to a lack of interpreters or availability of healthcare information in other languages. Often, women resorted to methods such as Google Translate, which is not reliable for translating medical terminology, jargon, or medications (75). For those with physical disabilities and accessibility needs, lack of relevant provision left some women feeling that they had lost their dignity (77). Staff were reported as unaware of service users’ accessibility requirements (having not read their file beforehand), or unaccommodating.

##### 3.4.5.2 Environmental factors

Social, economic, political, and religious aspects played roles in how women from underserved groups were treated in hospital (55). Societal prejudices and systemic discriminatory practices were reported to permeate personal care interactions (48). In systems where care is not free-to-access at the point-of-contact (such as in the United States), even with certain health insurance plans, financial constraints deterred women from seeking care until absolutely necessary (37).

### 3.5 Contribution to the candidacy framework

The 12 sub-themes of this meta-ethnography mapped onto all seven components of the Candidacy Framework, with two key observations: First, most sub-themes aligned with ‘navigation’ (*n* = 9) and ‘permeability of services’ (*n* = 6), which are joint and health system-level influences. Fewer connections were observed with other constructs: ‘adjudication’ (*n* = 6), ‘local production of candidacy’ (*n* = 3), ‘offers and resistance’ (*n* = 2), ‘appearances at health services’ (*n* = 5), and ‘identification’ (*n* = 3). Second, seven sub-themes only partially mapped to existing constructs: ‘authoritative knowledge struggle’, ‘stigma and mistrust’, ‘conceptualisation of pregnancy’, ‘lack of cultural competency’, ‘systems knowledge and social capital’, ‘structural inadequacies’, and ‘environmental factors’. This was especially true for those with a migrant background, suggesting the need for two additional constructs: *intercultural dissonance* (individual-level) and *hostile bureaucracy* (health system-level).

*Intercultural dissonance* encompasses additional barriers faced by those who are not native-born and experience a distinct difference in social norms and culture, medical and social knowledge and expectations, and language. Here, intergenerational relationships are altered by migration; for example, children (but not their parents) often speak (or speak more proficiently) the host country’s language, and are more familiar with the system, by virtue of having grown up there from a young age. As such, children take on more active roles in their parents’ healthcare decisions, such as acting as unofficial interpreters at care appointments, which may affect their parents’ ‘appearances at health services’ and ‘offers and resistance’ to care, as well as expose them to uncomfortable and potentially traumatic conversations and experiences.

*Hostile bureaucracy* sees migrant women often subject to discriminatory policies and precarious administrative practices in the host-country as compared to their home-country (86). These hostile, discriminatory immigration policies exist in most HICs, such as: restrictions on health coverage, welfare support, and right to rental properties; high visa application costs; and limits on qualifying employment. These policies, alongside negative societal attitude toward migrants and refugees, pose further barriers to integration into the host country, establishing a thriving life there, and accessing and engaging with healthcare. ‘Local production of candidacy’ is particularly diminished by these policies for migrant and refugee women.

**Figure 3:**
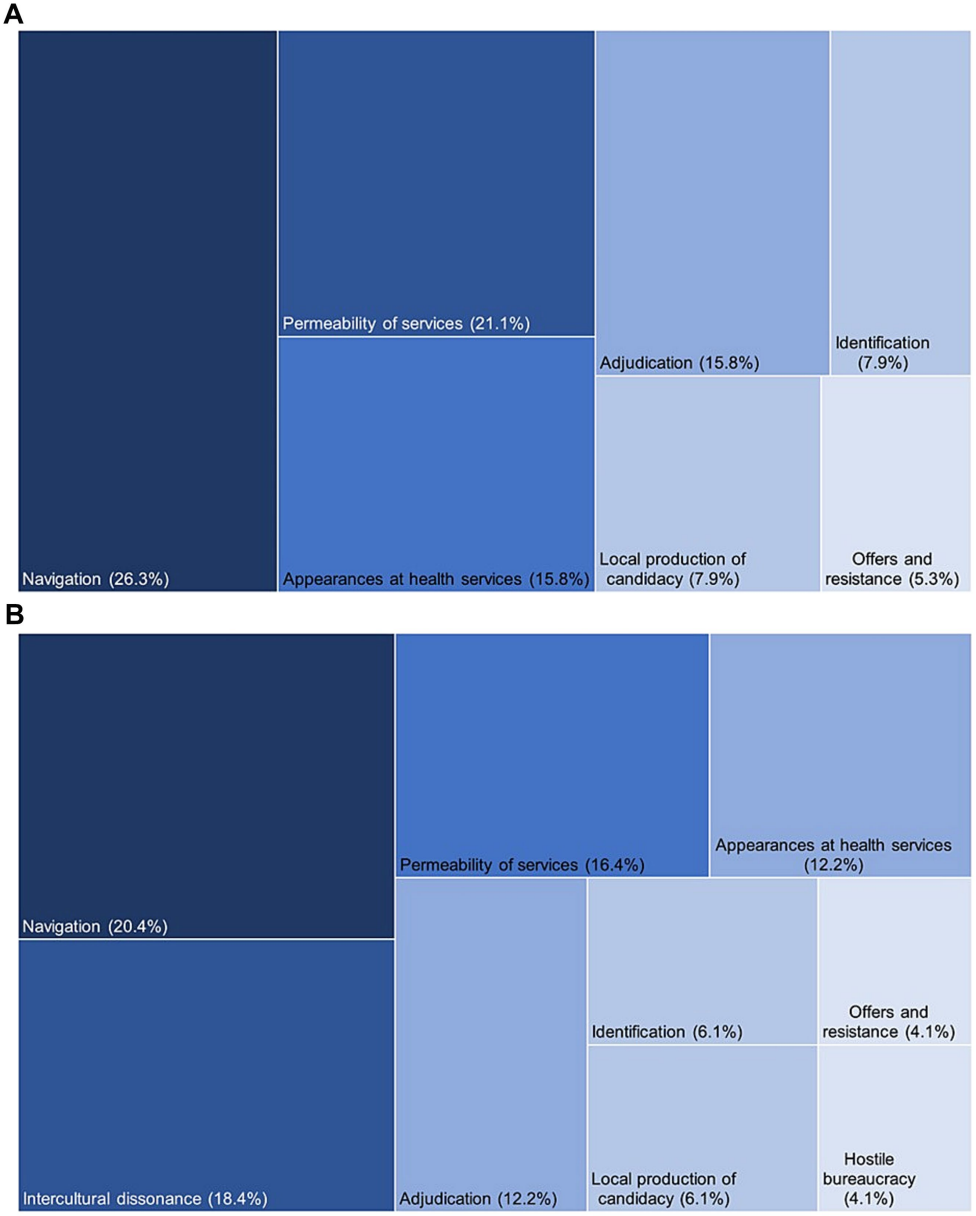
**(A,B)** Thematic contribution to the original and extended 7 + 2 Candidacy Framework, respectively.

Figure 3 shows a visual representation of the thematic contribution of our sub-themes to the original seven and extended 7 + 2 components of the Candidacy framework, respectively. For further details of the mapping process and candidacy framework components, see Supplementary Table S5, S6, respectively.

## 4 Discussion

### 4.1 Main findings

This systematic review identified 51 qualitative studies documenting, across 14 HICs, maternity care-seeking experiences of more than 1,300 women from minoritised and underserved groups. Twelve sub-themes emerged across five themes: (1) Loss of dignity, autonomy, and personhood; (2) Lack of informed choice and decision-making; (3) Trust *in* and relationships *with* HCPs; (4) Differences between healthcare systems and cultures; and (5) Systemic barriers. Experiences were largely negative. While sub-themes aligned with all seven components of the Candidacy Framework, most mapped to ‘navigation’, ‘permeability of services’, and ‘appearances at health services’, highlighting shared responsibility for improving care. Two new constructs—*intercultural dissonance* and *hostile bureaucracy*—emerged, particularly affecting migrants through altered intergenerational roles and exclusionary immigration policies. The meta-ethnography provided an analytic synthesis, rendering the theory: *‘Respect, informed choice, and trust enhances candidacy whilst differences in healthcare systems, culture, and systemic barriers have the propensity to diminish it’.*

### 4.2 Comparison with the literature

To our knowledge, this is the first systematic review focused exclusively on care-seeking experiences of diverse minoritised and underserved groups in HICs. Unlike our qualitative approach, most care-seeking research is quantitative, measuring attendance, visit frequency, or utilizations—often inconsistently defined (87) —and linking these to pregnancy outcomes. Frameworks like the social determinants of health (SDoH) model have been used to assess drivers of care-seeking, especially non-attendance (19, 26), including socio-cultural, political, and economic factors (19).

We build on a small number of reviews examining antenatal care among underserved groups (e.g., ethnic minorities, immigrants), which highlight complex barriers such as limited language skills, poor awareness of services, immigration and financial constraints, prior negative care experiences, and structural or organizational challenges (6, 88, 89).

This review found that care-seeking experiences were largely negative. A key issue was the lack of respectful treatment—dismissed concerns, unanswered questions, and unkind interactions—which left women feeling dehumanized (76, 79, 90). Prior research in LMICs shows that disrespectful care erodes trust and delays healthcare use (91, 92). For women with physical disabilities, inadequate attention to accessibility worsened this, leading to a loss of dignity (77). Stigma, discrimination, and insufficient information further undermined autonomy (56, 79). Quantitative studies also associate physical disability and one or more social risk factors to increased experiences of identity-related disrespect and reduced autonomy in maternity care (93, 94).

Our work demonstrates barriers to healthcare-seeking in pregnancy are jointly-driven, based on how frequently our sub-themes map to factors within the Candidacy Framework (19). Previous studies support this, identifying both system-level factors (e.g., organizational processes and system policies) (95–98) and individual-level factors (e.g., poor doctor-patient relationship (99), stigmatization (100), or being dismissed) (101). Improving care engagement for underserved women requires joint negotiation and co-production of services—such as the UK’s Maternity and Neonatal Voices Partnerships (MNVPs). The limited literature speaking to the constructs of the Candidacy Framework: ‘offers and resistance’ and ‘identification’ – joint- and individual-level factors – often places blame on women for low engagement attributing it to poor health literacy (58), problematising their language skils (102, 103), and further stigmatizing this already marginalised population.

A unique contribution of our study is the identification of *intercultural dissonance* and *hostile bureaucracy* as additions to the Candidacy Framework, reflecting the lasting and intergenerational effects of migration on care-seeking, including during pregnancy. Events of the last decade have emphasized the underserved nature of this population which has grown exponentially in the recent past due to various humanitarian crises. Differences in healthcare systems and cultures between ‘home’ countries and ‘host’ countries, significantly shape decisions about when and how to seek care, navigate services, and act on medical advice (70). This can lead to an *authoritative knowledge struggle*, where contradictory information (60, 104), may cause women to disengage from care altogether (45). Additionally, psychological research highlights how generational trauma and inherited knowledge influence wellbeing and behavior (105). A UK review of eight studies on asylum-seeking women identified barriers such as poor awareness of services, communication struggles, and stigma but did not explore how differing healthcare norms affect maternity experiences (98). Our meta-ethnographic approach, being generative and interpretive, was likely more attuned to these dynamics. Our proposed extension complements and builds on prior applications of the Candidacy Framework that have highlighted the unique challenges faced by migrants and minoritised groups in navigating healthcare (22, 106, 107). These have demonstrated how asylum seekers encounter systemic exclusions and bureaucratic hurdles (106, 107) that cannot be fully explained by the existing seven constructs (106, 107), and how cultural differences and intergenerational dynamics shape access (22). By adding the constructs of *intercultural dissonance* and *hostile bureaucracy*, our work extends this trajectory, offering conceptual tools that better capture the multi-generational, structural, and cultural dimensions of exclusion in perinatal care-seeking among underserved women.

While our synthesis identifies common themes in migrant women’s experiences, the 13 HICs represented (e.g., United Kingdom, United States, Saudi Arabia) vary widely in healthcare models, migrant entitlements, and cultural expectations. For instance, healthcare fees in the US or restrictions on undocumented migrants in Europe may intensify systemic barriers compared to countries with universal access. These structural and cultural differences affect the transferability of findings, particularly in relation to how barriers manifest and are addressed. Tools such as the WHO Health Financing Progress Matrix or Migrant Integration Policy Index which examines how well a country’s health financing policies align with achieving universal coverage (108), and policies to integrate migrants and other marginalised groups into society (109) respectively show marked difference between our 14 included countries. Only two countries (Sweden and Canada) ranked highly in both assessments. Such factors are likely to influence care-seeking behavior, shaping the relevance of our findings.

Additionally, while our sample includes a diverse group of marginalised communities with several complex social risk factors, only two (55, 81) of the 51 studies consider the idea of intersectionality. It has now been well-established that individuals with multiple marginalised identities face compounded barriers to care access and utilizations (110), and future research is crucial to understanding these unique intersections of disadvantage.

### 4.3 Strengths, limitations, and future directions

A key strength of our review is the use of meta-ethnography, allowing us to build on themes from individual studies—all of which were of moderate-to-high quality—and identify gaps in existing theory. Notably, we highlight the multi-generational impact of immigration on care-seeking as a missing component of the Candidacy Framework, supporting its expansion. Our focus on women’s care-seeking excluded perspectives of fathers, partners, non-gestational parents, providers, and policymakers. While we observed similarities across groups and countries, we may have missed group-specific or system-level differences, which we plan to explore further. A planned sub-group analysis on the pandemic’s impact was not possible due to limited studies; questions remain on how service reconfigurations and misinformation shaped care-seeking during this time and will be explored by is in future qualitative work. Future research should also examine the roles of families, professionals, and health systems, and empirically validate the proposed construct of intercultural dissonance.

## 5 Conclusion

In HICs, maternity care-seeking is a joint responsibility between service-users and service-providers. As such, interventions to remove barriers to care-seeking should be co-produced through collaborative means between stakeholders. Efforts to improve utilization *of,* and engagement *with,* antenatal care services should prioritize alleviating system-level barriers. We suggest an expansion of the Candidacy Framework to include two further dimensions which reflect the multigenerational effect of migration on care experience and the often hostile and precarious bureaucratic environment in which women find themselves when attempting to seek maternity care.

## Data Availability

The original contributions presented in the study are included in the article/Supplementary material, further inquiries can be directed to the corresponding author.
